# *Syn*- and *anti*-rotamers of the *ortho*-stereoisomer [Pt{(*o*-BrC_6_F_4_)N(CH_2_)_2_NEt_2_}Cl(py)]

**DOI:** 10.1107/S2053229625006837

**Published:** 2025-08-21

**Authors:** Ruchika Ojha, Alan M. Bond, Peter C. Junk, Glen B. Deacon

**Affiliations:** a School of Chemistry, Monash University, VIC 3800, Australia; b School of Science, STEM College, RMIT University, Melbourne, VIC 3000, Australia; c College of Science & Engineering, James Cook University, Townsville, Qld 4811, Australia; University of North Texas at Dallas, USA

**Keywords:** crystal structure, platinum anticancer agent, *syn* and *anti* rotamers, agostic interactions, synchrotron

## Abstract

The crystal structure of the *ortho*-isomer *trans*-[*N*-(2-bromo-3,4,5,6-tetrafluorophenyl)-*N*′,*N*′-diethylethane-1,2-diaminato(1−)]chloridopyridineplatinum(II), [PtBr_0.1_(C_12_H_14_BrF_4_N_2_)Cl_0.9_(C_5_H_5_N)][PtBr_0.4_(C_12_H_14_BrF_4_N_2_)Cl_0.6_(C_5_H_5_N)] or [Pt{(*o*-BrC_6_F_4_)N(CH_2_)_2_NEt_2_}Cl(py)], **1*****o***, revealed *syn* and *anti* rotamers in a 1:1 ratio in the solid state. **1*****o*** crystallizes in the centrosymmetric space group *P*1. The Pt-coordinated Cl ligand exhibits partial occupancy with Br, predominantly in the *syn*-rotamer. Notably, agostic inter­actions are observed between the Pt centre and a H atom of one of the ethyl groups. The *ortho*-isomer **1*****o*** was successfully isolated as a side product from the reaction of [Pt{H_2_N(CH_2_)_2_NEt_2_}Cl_2_], Tl_2_CO_3_ and C_6_F_5_Br. While the *para*-isomer [Pt{(*p*-BrC_6_F_4_)N(CH_2_)_2_NEt_2_}Cl(py)], **1*****p***, is the main product, the higher solubility of **1*****o*** facilitates its isolation.

## Abstract 2

Greater solubility facilitated the isolation of the *ortho*-isomer from a CO_2_ elimination reaction that primarily produces the *para*-isomer [Pt{(*p*-BrC_6_F_4_)N(CH_2_)_2_NEt_2_}Cl(py)]. X-ray crystallography identified the *syn* and *anti* rotamers of [Pt{(*o*-BrC_6_F_4_)N(CH_2_)_2_NEt_2_}Cl(py)] in a 1:1 ratio in the solid state.

## Introduction

**Figure 1:**
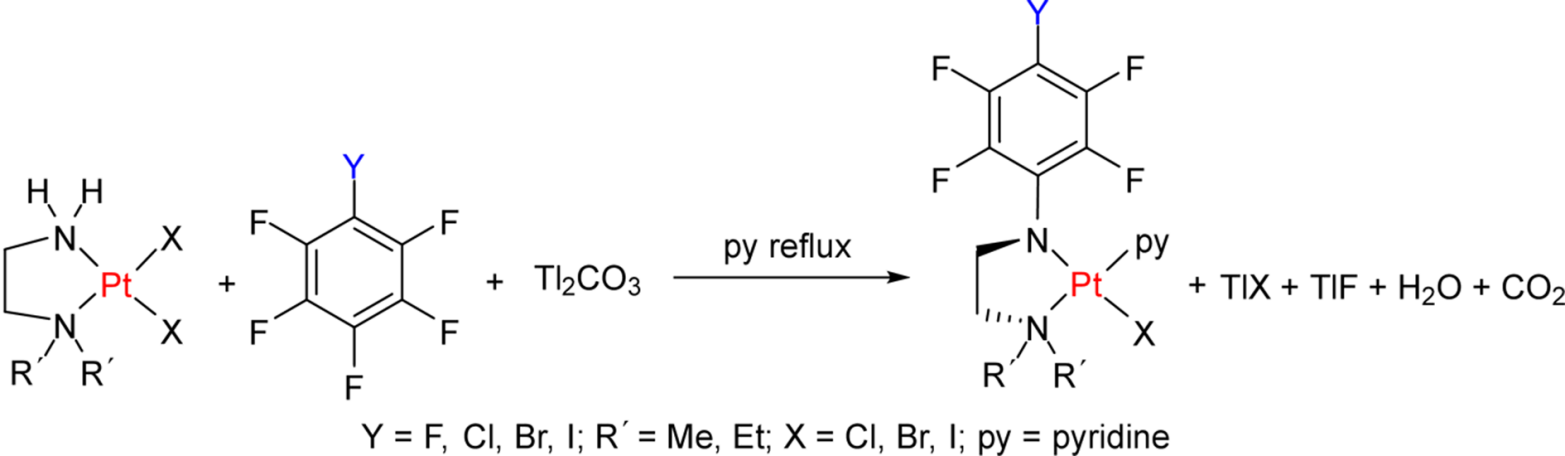
General synthesis of [Pt{*R*N(CH_2_)_2_N*R*′_2_}*X*(py)].

Polyfluoroaryl-substituted organo­amido­platinum(II) com­plexes [Pt{*R*N(CH_2_)_2_N*R*′_2_}*X*(py)] [*R* = *p*-*Y*C_6_F_4_ (*Y* = F, Cl, Br or I), CH_3_, *etc*.; *R*′ = Me or Et; *X* = Cl, Br or I; py = pyridine] have been shown to have good *in vitro* and modest *in vivo* activity (Talarico *et al.*, 1999 ▸; Ojha *et al.*, 2021 ▸) against a number of tumour cells. They are conveniently prepared by reaction of [Pt*X*_2_(NH_2_CH_2_CH_2_N*R*′_2_)] with thallium(I) car­bon­ate (or K_2_CO_3_ in some cases) and a polyfluoro­arene, *R*F, in boiling pyridine (Fig. 1 ▸) (Battle *et al.*, 2010 ▸; Ojha *et al.*, 2015 ▸). The CO_2_ generated from Tl_2_CO_3_ during the reaction was trapped as BaCO_3_ by a Ba(OH)_2_ solution, and the yield of CO_2_ was measured gravimetrically.

One step in the com­plex CO_2_ elimination reaction paths is nucleophilic substitution of F on the polyfluoro­benzene, *R*F, by the –NH_2_ group, plausibly partially deprotonated by the carbonate group. The substitution pattern of the major products (Battle *et al.*, 2010 ▸; Buxton *et al.*, 1988 ▸; Deacon *et al.*, 1991 ▸) corresponds to that (*para* to substituent *Y*), as observed in the nucleophilic substitution of polyfluoro­arenes (Chambers *et al.*, 1974 ▸, 1977 ▸; Chambers, 2004 ▸). Although the ^19^F NMR spectra of crude reaction products sometimes suggested that the reactions were not entirely regiospecific, simple recrystallization usually gave isomerically pure products (Battle *et al.*, 2010 ▸; Buxton *et al.*, 1988 ▸; Deacon *et al.*, 1991 ▸). However, the reaction of [PtCl_2_(en)] (en is ethylenediamine), Tl_2_CO_3_ and 2-bromo-1,3,4,5-tetra­fluoro­benzene in pyridine gave isomers with the N atom *para* to both H and Br. Only the former was isolated, the latter being identified spectroscopically in the reaction mixture (Battle *et al.*, 2010 ▸).

**Figure 2:**
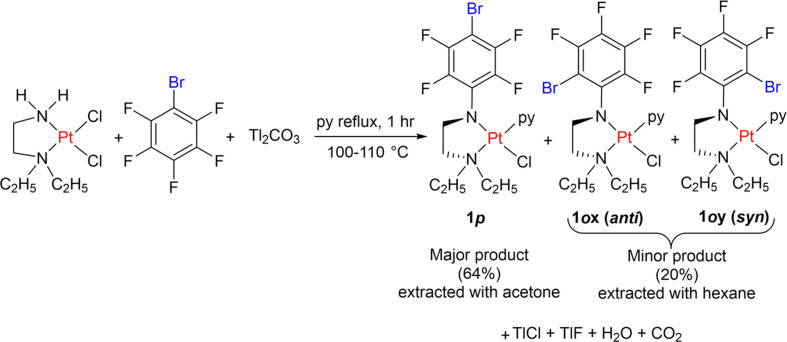
Carbon dioxide elimination reaction for the synthesis of **1*****p*** and **1*****ox***/**1*****oy***.

We have recently reported anti­cancer activity (Ojha *et al.*, 2021 ▸), chemical oxidation (Ojha *et al.*, 2023 ▸) and the synthesis of [Pt{(*p*-BrC_6_F_4_)N(CH_2_)_2_NEt_2_}Cl(py)], **1*****p*** (with *X* = Cl, *R*′ = Et and *R* = *p*-BrC_6_F_4_), in 64% yield by reaction between [PtCl_2_{H_2_N(CH_2_)_2_NEt_2_}], Tl_2_CO_3_ and C_6_F_5_Br in pyridine (Fig. 2 ▸) (Ojha *et al.*, 2015 ▸). During this study, it was noticed that the hexane washings of the crude product (to remove adherent pyridine) had a yellow colour. We have now investigated the source of the colour and have isolated and crystallized the *ortho*-isomer, [Pt{(*o*-BrC_6_F_4_)N(CH_2_)_2_NEt_2_}Cl(py)], **1*****o***. This has been identified by X-ray crystallography, employing synchrotron radiation, and found to crystallize as a 1:1 mixture of the *syn* (**1*****ox***) and *anti* (**1*****o*****y)** rotamers (with respect to the *o*-Br and Pt—Cl positions) in the asymmetric unit.

## Experimental

### General

NMR spectra were recorded in deuterated acetone with a Bruker DPX 400 spectrometer supported by *Top Spin* NMR software on a Windows NT workstation. CFCl_3_ and tetra­methyl­silane (TMS) were used for the inter­nal calibration of the ^19^F NMR and ^1^H NMR spectra, respectively. IR spectra were recorded on a Perkin–Elmer 1600 FT–IR spectrophotometer as Nujol and hexa­chloro­butadiene (HCB) mulls between NaCl plates or recorded with an Agilent Cary 630 attenuated total reflectance (ATR) spectrometer in the range 4000–600 cm^−1^.

### X-ray crystallography

Crystal data, data collection and structure refinement details are summarized in Table 1 ▸. X-ray diffraction data obtained from single crystals of **1*****ox***/**1*****oy*** were collected at a wavelength of λ = 0.712 Å using the MX1 beamline at the Australian Synchrotron, Victoria, Australia, with a *Blue Ice* (McPhillips *et al.*, 2002 ▸) GUI, using the same method as mentioned in the *Experimental* section of Ojha *et al.* (2015 ▸). Data were processed with the *XDS* (Kabsch, 1993 ▸) software package. Single crystals were loaded onto a fine glass fiber or cryoloop using hydro­carbon oil, with the collection kept at 123 K using an Oxford Cryosystems open-flow N_2_ Cryostream. The pro­gram *OLEX2* (Dolomanov *et al.*, 2009 ▸) was used as the graphical inter­face. H atoms attached to C atoms were placed in calculated positions and allowed to ride on the atom to which they were attached.

### Isolation of *ortho*-isomers [Pt{(*o*-BrC_6_F_4_)NCH_2_CH_2_NEt_2_}Cl(py)], 1*ox*/1*oy*

After completion of the typical synthesis of **1*****p*** by a CO_2_ elimination reaction (Ojha *et al.*, 2015 ▸), pyridine was removed under vacuum until dryness. Hexane was added to remove traces of residual pyridine and decanted. The major product **1*****p*** was extracted with acetone from the remaining solid, as reported earlier. The decanted hexane was yellow–orange, rather than colourless, indicating that it had not just removed the remaining pyridine, but possibly an isomer.

To isolate and crystallize the isomers, some acetone was added to the deca­nted solution. Crystals of **1*****ox***/**1*****oy*** suitable for structure determination were obtained by slow evaporation of the solvent. **1*****ox*** and **1*****oy*** are present in a 1:1 ratio. Apart from the X-ray data, the integrations for ^1^H resonances measured in (CD_3_)_2_CO show **1*****ox***/**1*****oy*** in a 1:1 ratio.

Metallic yellow–orange blocks (yield: 0.130 g, 20%). ^19^F NMR [(CD_3_)_2_CO]: δ −140.6 (*d*, 2F, F3), −151.2 (*d*, 2F, F6), −160.9 (*t*, 2F, F5), −171.1 (*m*, 2F, F4). ^1^H NMR [(CD_3_)_2_CO]: δ 1.53 (*t*, ^3^*J*_H,H_ = 7.15 Hz, 12H, NCH_2_C**H_3_**), 2.48 (*t*, with ^195^Pt–H satellites, ^3^*J*_H,H_ = 6, ^3^*J*_H,Pt_ = 30 Hz, 4H, C**H_2_**NEt_2_), 2.80 (*m*, 4H, NC**HA**HBCH_3_), 3.34 [*m*, 8H, made up of 4H C**H_2_**N(*p*-BrC_6_F_4_) and 4H NC**HB**HACH_3_], 7.09 [*t*, ^3^*J*_H,H_ = 7 Hz, 2H, **H3,5**(py)], 7.15 [*t*, 2H, ^3^*J*_H,H_ = 7 Hz, **H3,5**(py)], 7.65 [*tt*, ^3^*J*_H,H_ = 7, ^4^*J*_H,H_ = 1 Hz, 1H, **H4** (py)], 7.70 [*tt*, ^3^*J*_H,H_ = 7, ^4^*J*_H,H_ = 1 Hz, 1H, **H4** (py)], 8.50 [*d* with ^195^Pt–H satellites, ^3^*J*_H,H_ = 5, ^3^*J*_H,Pt_ = 36 Hz, 2H, **H2,6**(py)], 8.54 [*d* with ^195^Pt–H satellites, ^3^*J*_H,H_ = 5, ^3^*J*_H,Pt_ = 36 Hz, 2H, **H2,6**(py)]. IR (cm^−1^): 2960 (*w*), 2922 (*w*), 2853 (*w*), 1654 (*w*), 1618 (*w*), 1607 (*b*), 1458 (*s*), 1450 (*s*), 1375 (*m*), 1345 (*w*), 1258 (*s*), 1208 (*m*), 1133 (*s*), 1073 (*s*), 1014 (*s*), 962 (*s*), 898 (*m*), 875 (*m*), 794 (*s*), 765 (*s*), 691 (*s*).

## Results and discussion

The *ortho*-isomer **1*****o*** was preferentially isolated due to its markedly greater solubility in hexane com­pared to the *para*-isomer **1*****p***. The synthesis predominantly afforded **1*****p*** (Ojha *et al.*, 2015 ▸) by a CO_2_ elimination reaction (Fig. 2 ▸). A subsequent hexane washing, intended to remove residual pyridine, unexpectedly exhibited a yellow–orange coloration. This observation suggested the presence of an additional platinum-containing species, which could be a different isomer, rather than merely solvent. Therefore, it was investigated further, and slow evaporation of the hexane washing enabled the isolation of the *ortho*-isomer [Pt{(*o*-BrC_6_F_4_)NCH_2_CH_2_NEt_2_}Cl(py)], **1*****o***, which was considerably more soluble in the low-polarity solvent hexane (with a trace of pyridine) than **1*****p***. The *ortho*-isomer **1*****o*** crystallized as a 1:1 mixture of the *anti* (**1*****ox***) and *syn* (**1*****oy***) rotamers in the asymmetric unit. This procedure facilitates isolation of the pure *para*-isomer (**1*****p***) as the major product from the reaction mixture.

### Characterization of 1*o*

The initial identification of **1*****o*** was *via*^1^H and ^19^F NMR spectroscopy in (CD_3_)_2_CO. The coordination of pyridine and the amide ligand to platinum was evident from the observation of ^3^*J*(^195^Pt,H) satellites (^195^Pt isotope, nuclear spin *I* = 1/2, natural abundance = 33.8%) on the signals of the H2,6(pyridine) and CH_2_(N-eth­yl) protons, with the coupling constants ^3^*J*(Pt,H2,6-py) and ^3^*J*(Pt,CH_2_-N) having values (36 and 30 Hz, respectively) similar to those (35 and 28 Hz) observed for **1*****p*** (Ojha *et al.*, 2015 ▸). Other ^1^H chemical shifts and integrations, which are similar to those of **1*****p***, are consistent with the com­position of **1*****o***.

Evidence for the proposed polyfluoro­phenyl substitution pattern comes from ^19^F NMR spectroscopy. Four equal-intensity ^19^F resonances indicate either a *m*-BrC_6_F_4_ or an *o*-BrC_6_F_4_ group com­pared with two for **1*****p*** (Fig. S1 for the F-atom numbering system). Chemical shift calculations {based on substituent chemical shifts for Br (Bruce, 1968 ▸; Ando & Matsuura, 1995 ▸), for *o*- and *m*-[N(–CH_2_)Pt] groups derived from **1*****p*** (Ojha *et al.*, 2015 ▸), and for *p*-[N(CH_2_–)Pt] derived from several [Pt{C_6_F_5_NCH_2_CH_2_NEt_2_}*X*(py)] com­plexes (Deacon *et al.*, 1991 ▸)] clearly support the presence of an *o*-BrC_6_F_4_ substituent in **1*****o***, and com­pares well with the observed chemical shift (Table 2 ▸). The ^19^F NMR spectrum is provided in the supporting information (Fig. S2) and shows the same chemical shifts for **1*****ox*** and **1*****oy***. In the ^1^H NMR spectrum, the pyridine resonances in **1*****ox*** and **1*****oy*** appear 0.1 ppm apart, as shown in Fig. S3, and show **1*****ox*** and **1*****oy*** in a 1:1 ratio.

The unequivocal identification of **1*****o*** was provided by X-ray crystallography. The crystallographic data differ considerably from those of **1*****p*** (Table 1 ▸). **1*****o*** crystallizes in the triclinic space group *P*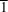 with the rotamers **1*****ox*** and **1*****oy*** (Fig. 2 ▸) in the asymmetric unit (Table 1 ▸). In **1*****ox***, Br and Cl are *anti* with a Br—Pt—Cl angle of 156.86 (6)°, whereas in **1*****oy***, they are in a *syn* disposition with a Br—Pt—Cl angle of 113.61 (8)°.

In the proposed mechanism, initially, both chloride ligands on Pt are replaced by pyridine. Due to the hy­dro­gen bonding between –NH_2_ and CO_3_^2−^, a lone-pair character is generated on the N atom and initiates nucleophilic substitution in the polyfluoroaryl ring (Deacon *et al.*, 1998 ▸), as shown in Scheme S1 in the supporting information.

**Figure 3:**
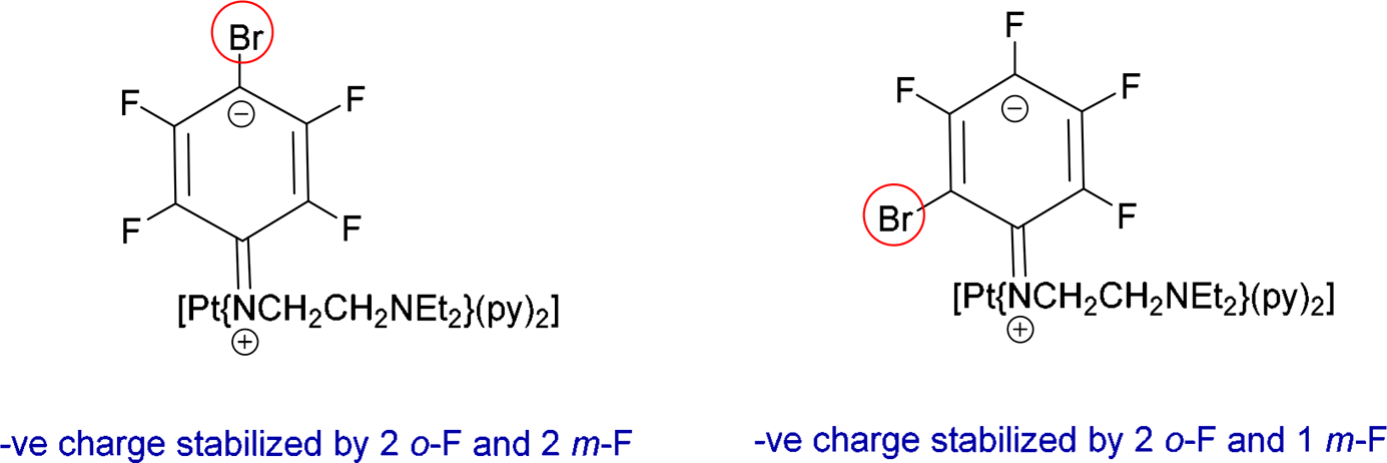
The Meisenheimer inter­mediates formed during the formation of **1*****p*** (left) and **1*****o*** (right).

The Meisenheimer inter­mediates involved in the formation of **1*****p*** and **1*****o*** are depicted in Fig. 3 ▸. In the case of **1*****p***, the negative charge generated during the nucleophilic substitution of the polyfluoroaryl ring is stabilized by two *ortho*- and two *meta*-fluorines, relative to the site of substitution (see Scheme S2 in the supporting information). Similarly, the formation of the *ortho*-Br isomers is also feasible because the negative charge in the Meisenheimer inter­mediate (Fig. 3 ▸) is located *para* and *ortho* to the site of substitution. This causes the positions *ortho* and *para* to Br to be electron deficient and thus susceptible to nucleophilic attack (Scheme S2). The negative charge in the Meisenheimer inter­mediate is stabilized by two *o*-F and two *m*-F atoms in **1*****p***, and by two *o*-F and one *m*-F atom in **1*****o***.

The displacement of the pyridine ligand *trans* to the amide group by the chloride ion gives the target com­pound (see Scheme S1 in the supporting information). This regiospecificity is obtained as the *trans* effect of the –N(*p*-BrC_6_F_4_) N atom is greater than that of the –NEt_2_ N atom, in line with the *trans*-influence values from platinum–H coupling constants (Buxton *et al.*, 1988 ▸).

In **1*****ox***, the Cl ligand coordinated to the Pt atom has a shared occupancy with Br, *cf*. 0.59 (1):0.41 (1), yielding 0.59 Cl and 0.41 Br, while for **1*****oy***, the Cl remains the major occupant, with 0.91 (1) Cl and a slight sharing 0.09 (1) with Br. The Br atom is derived from C_6_F_5_Br. It has previously been shown that some elimination of Br occurs during the oxidation of **1*****p*** by hy­dro­gen peroxide (Ojha *et al.*, 2021 ▸), and replacement of chloride coordinated to Pt by bromide is consistent with the stability constants for soft metals (Ault *et al.*, 1977 ▸).

**Figure 4:**
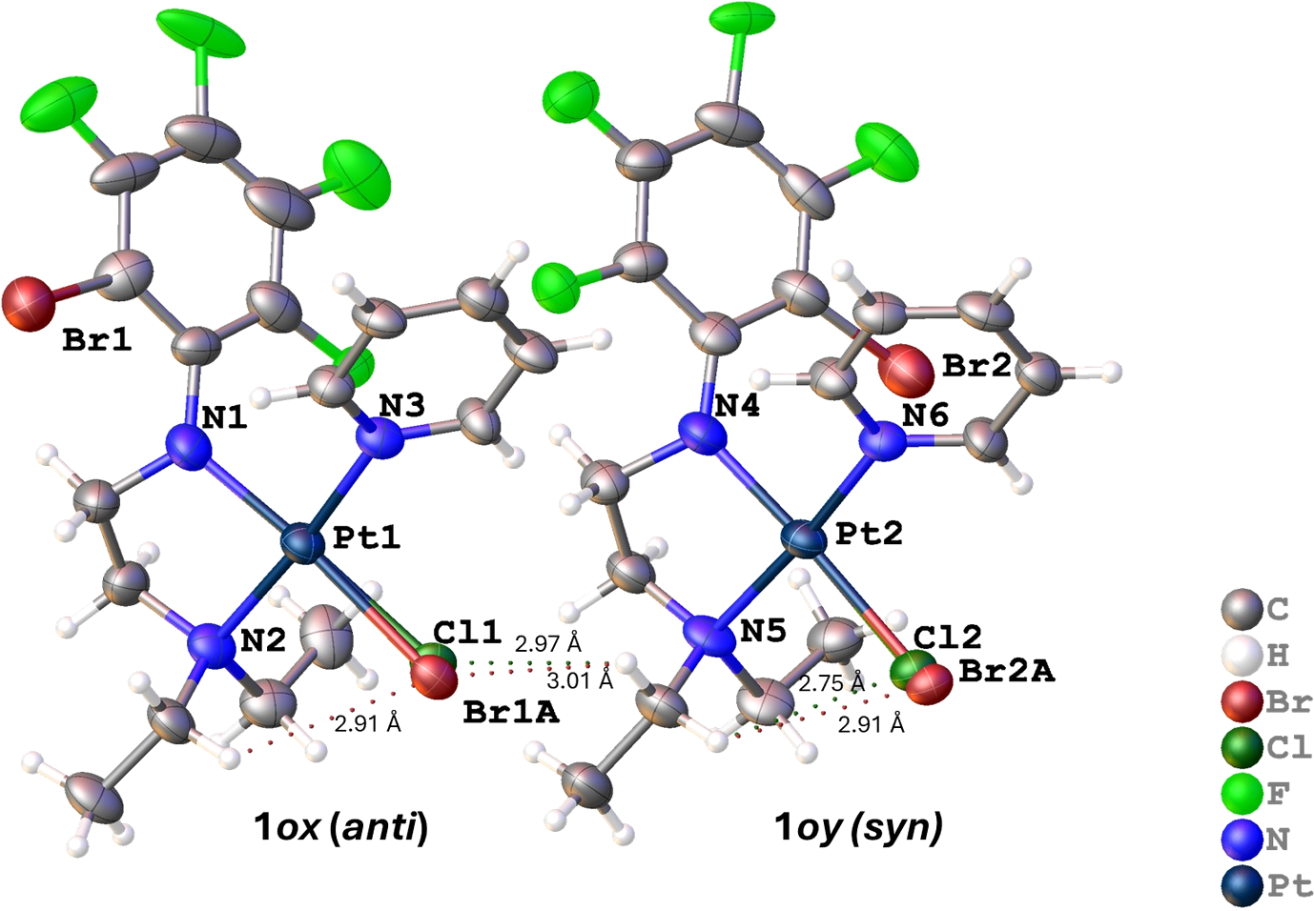
The mol­ecular crystal structures of rotamers **1*****ox*** (*anti*) and **1*****oy*** (*syn*) cocrystallized in a single unit cell, showing 50% probability displacement ellipsoids.

The mol­ecular structure of **1*****o*** shows that the Pt atom is coordinated in a square-planar array by a chelating {(*o*-BrC_6_F_4_)NCH_2_CH_2_NEt_2_}^−^, pyridine and chloride ligands, with the chloride ligand being *trans* to the amide N atom and pyridine being *trans* to the amine group (Fig. 4 ▸). Thus, it is a *trans*-isomer in terms of the positions of the like-charged donor atoms. Selected bond lengths and angles for **1*****ox***/**1*****oy*** are given in Table 3 ▸ and com­pared with those of **1*****p***. In general, the values for **1*****ox***/**1*****oy*** and **1*****p*** agree within or near the 3 e.s.d. level. However, the Pt—Cl bond of **1*****ox*** is longer than that of **1*****oy*** or **1*****p***, owing to the shared Cl/Br occupancy. This is not a steric effect as the bond does not appear crowded. Supra­molecular effects need to be considered. The Pt—N bond lengths follow the sequence Pt—N(amide) < Pt—N(py) < Pt—N(Et_2_) (Table 3 ▸), as was also observed for **1*****p***. Most bond angles around the Pt centre are 90°, with the smallest being the bite angles of 84.1° for **1*****ox*** and 83.5° for **1*****oy***. The –NCH_2_—CH_2_N– sawhorse backbone is crooked, as seen in **1*****p*** and other com­pounds of this class (Deacon *et al.*, 1991 ▸; Ojha *et al.*, 2016 ▸).

**Figure 5:**
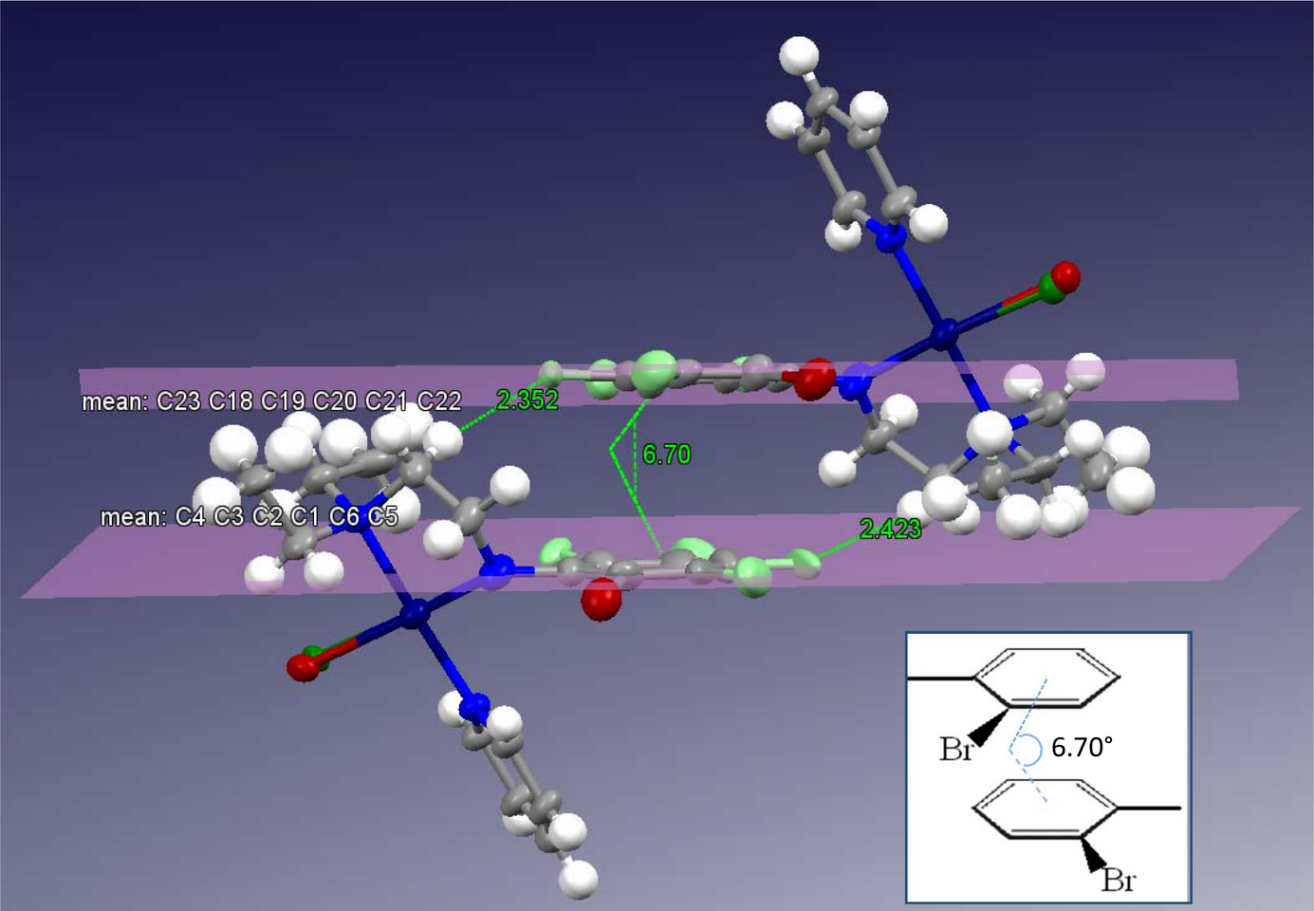
The π–π inter­action between the two polyfluoroaryl rings of two mol­ecules with an angle of 6.703 (3)°, where the mirror image is rotated by 180° and the symmetry code is (−*x* + 1, −*y*, −*z* + 1). The inset shows the *ortho*-Br atoms.

Intra­molecular hy­dro­gen bonding in **1*****ox*** is observed as (NEt_2_)H⋯Br, with an H⋯Br distance of 2.91 (2) Å, while **1*****oy*** displayed an (NEt_2_)H⋯Br inter­action of 2.91 (4) Å and an (NEt_2_)H⋯Cl inter­action of 2.754 (9) Å (Fig. 4 ▸). Inter­molecular hy­dro­gen bonding between the **1*****ox*** Cl/Br atoms and the H(NEt_2_) atom of **1*****oy***, with an (NEt_2_)H⋯Br distance of 3.093 (19) Å and an (NEt_2_)H⋯Cl distance of 2.97 (3) Å, was also observed. A π–π inter­action between the two polyfluoroaryl rings is present (but not between py rings) and, in this arrangement, the polyfluoroaryl rings are not parallel but have an inter­planar angle of 6.703 (3)°, as shown in Fig. 5 ▸. The *ortho*-Br atoms of both mol­ecules are on the same side (as shown in the inset of Fig. 5 ▸), resulting in significant steric hindrance on one side. Consequently, the polyfluoroaryl rings are tilted at an angle of 6.703 (3)° to reduce the steric hindrance. The inter-centroid distance is 3.7969 (10) Å and the rings are offset by 1.7513 (15) Å, as was also observed for other similar com­pounds (Ojha *et al.*, 2018 ▸). On the other hand, in **1*****p***, a π–π inter­action was observed between two pyridine rings, and not between polyfluoroaryl rings.

**Figure 6:**
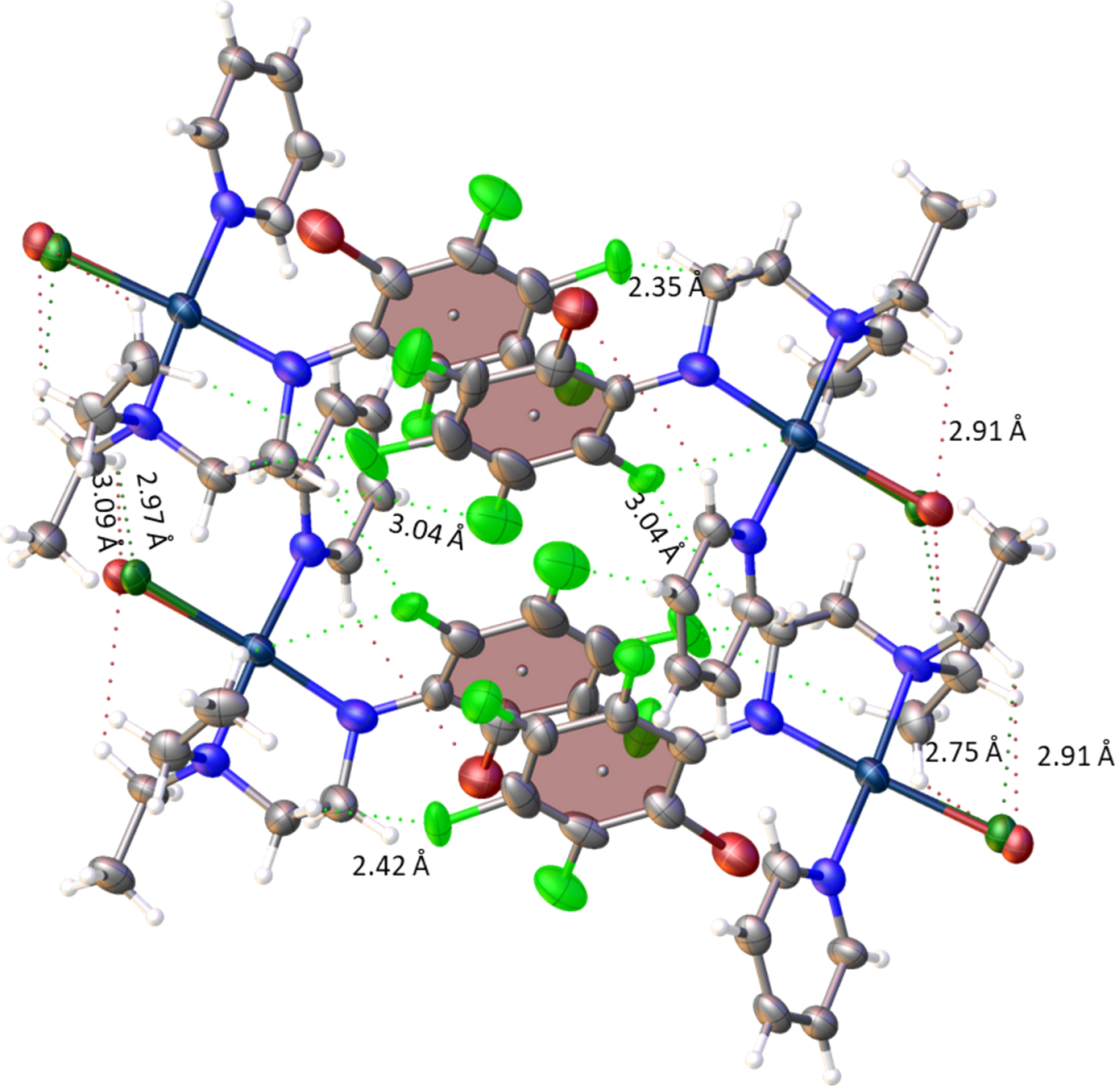
The crystal packing in **1*****ox***/**1*****oy***, showing the π–π inter­actions between the two polyfluoroaryl rings of **1*****ox*** and **1*****oy***, and inter- and intra­molecular hy­dro­gen bonding.

The π–π inter­action is further anchored by strong inter­molecular hy­dro­gen bonding between the *para*-F atom of **1*****ox*** with a methyl­ene H of the ligand backbone of **1*****oy***, and *vice versa*, as shown in Fig. 6 ▸, with H⋯F distances of 2.352 (7) and 2.424 (10) Å. Additionally, com­paratively weak inter­actions, such as between the *para*-F atom of **1*****ox*** with a methyl H atom of the NEt_2_ group, with an H⋯F distance of 3.012 (8) Å, and a very weak inter­action between the *ortho*-F atom of **1*****ox*** and a methyl­ene H atom of the ligand backbone of **1*****oy***, with a H⋯F distance of 3.353 (11) Å, further stabilize the π–π inter­action.

**Figure 7:**
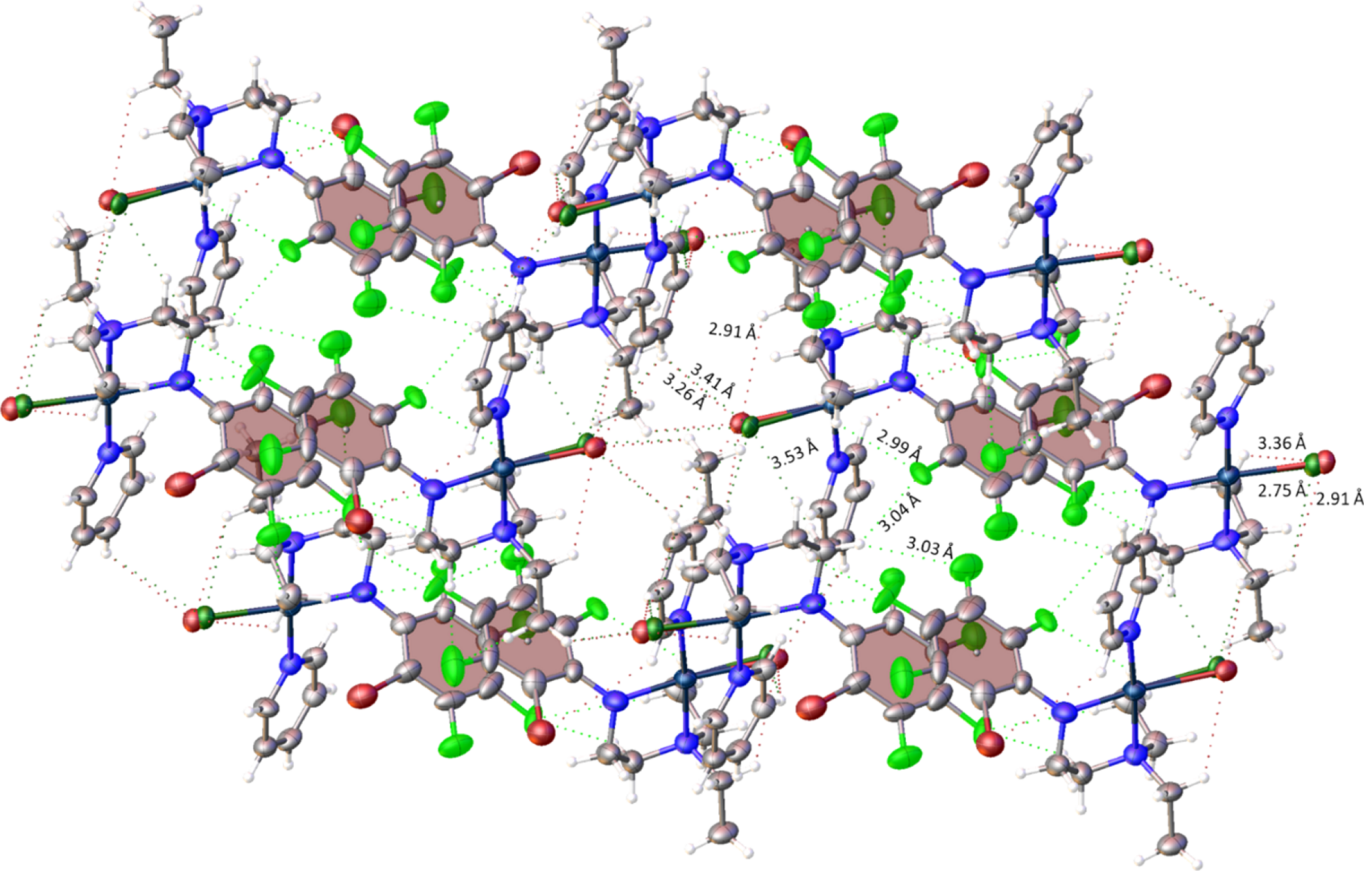
The crystal packing in **1*****ox***/**1*****oy***, showing H⋯Cl inter­actions for **1*****ox*** and C⋯H inter­actions for **1*****oy*** as inter­molecular hy­dro­gen bonding.

In the **1*****ox***/**1*****oy*** isomers, one Et group makes an agostic inter­action with Pt; the Pt⋯H(CH_3_) distance is 2.8043 (11) Å and the bond angles are 118.8 (3)° for H—Pt—N(py), 103.8 (3)° for H—Pt—N(C_6_F_5_) and 65.3 (3)° for H—Pt—N(Et)_2_ in **1*****ox***, and the Pt⋯H(CH_3_) distance is 3.0032 (11) Å and the bond angles are 120.0 (3)° for H—Pt—N(py), 106.7 (3)° for H—Pt—N(C_6_F_5_) and 66.1 (3)° for H—Pt—N(Et)_2_ in **1*****oy***. The *ortho*-F atom of **1*****ox*** makes an intra­molecular hy­dro­gen-bonding contact with a methyl H atom of –N(Et_2_), which exhibits an agostic inter­action with Pt, with an H⋯F distance of 2.994 (7) Å (Fig. 7 ▸). These rotamers display the entire network of supra­molecular inter­actions, as illustrated in Fig. 7 ▸. The *p*-H(py) atom of **1*****ox*** is anchored by inter­molecular hy­dro­gen bonding with the Cl/Br atom of the two adjacent **1*****ox*** mol­ecules, with H⋯Br distances of 3.050 (16) and 3.26 (2) Å, and H⋯Cl distances of 3.15 (2) and 3.41 (3) Å (see Fig. 7 ▸). Similarly, the *m*-H(py) atom is involved in hy­dro­gen bonding with the Cl/Br atom of another **1*****ox*** mol­ecule, with a H⋯Br distance of 2.78 (4) Å and a H⋯Cl distance of 2.578 (8) Å.

Inter­molecular F⋯H hy­dro­gen bonding of two adjacent **1*****ox*** mol­ecules, with an F⋯H distance of 2.915 (9) Å, was observed between the *m*-F atom of the polyfluoroaryl ring and a methyl H of the Et group (–NEt_2_), the one not showing the agostic inter­actions with Pt (Fig. 7 ▸). These supra­molecular inter­actions may facilitate the docking of the drug and reinforce the nucleobase–Pt inter­actions.

## Conclusion

Further examination of the products of the reaction between [PtCl_2_{H_2_N(CH_2_)_2_NEt_2_}], Tl_2_CO_3_ and bromo­penta­fluoro­benzene in refluxing pyridine has revealed that, in addition to the major product, [Pt{(*p*-BrC_6_F_4_)N(CH_2_)_2_NEt_2_}Cl(py)], *i.e.***1*****p***, a significant amount of the *ortho*-stereoisomer, [Pt{(*o*-BrC_6_F_4_)N(CH_2_)_2_NEt_2_}Cl(py)], *i.e.***1*****o***, can also be isolated, taking advantage of the much higher solubility of **1*****o***. The new regioisomer, which was characterized by synchrotron X-ray crystallography, crystallizes as a 1:1 mixture of two rotameric isomers, *i.e.***1*****ox*** and **1*****oy***, according to whether the Br substituent and the Cl ligand are in an *anti* (in **1*****ox***) or *syn* (in **1*****oy***) disposition. The ^1^H and ^19^F NMR spectra in (CD_3_)_2_CO are consistent with the structural assignment.

## Supplementary Material

Crystal structure: contains datablock(s) I, global. DOI: 10.1107/S2053229625006837/yd3064sup1.cif

Structure factors: contains datablock(s) I. DOI: 10.1107/S2053229625006837/yd3064Isup2.hkl

Numbering schemes, reation pathway, resonance structures and <sup>19</sup>F and <sup>1</sup>H NMR spectra. DOI: 10.1107/S2053229625006837/yd3064sup3.pdf

CCDC references: 2481234, 2477306

## Figures and Tables

**Table 1:** Crystallographic data for the mol­ecular structures of **1*ox***/**1*oy*** and com­parison with **1*p***

	*ortho*-**1*ox***/**1*oy***	**1*p*** (Ojha *et al.*, 2015 ▸)
Empirical formula	C_34_H_38_Br_2.5_Cl_1.5_F_8_N_6_Pt_2_	C_17_H_19_BrClF_4_N_3_Pt
Formula weight	1321.39	651.78
Crystal system	Triclinic	Monoclinic
Space group	*P* 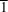	*P*2_1_/*c*
*a* (Å)	9.4810 (19)	10.960 (2)
*b* (Å)	14.656 (3)	11.961 (2)
*c* (Å)	15.094 (3)	15.224 (3)
α (°)	75.02 (3)	90
β (°)	74.62 (3)	98.46 (3)
γ (°)	86.28 (3)	90
*V* (Å^3^)	1953.5 (8)	1974.0 (7)
*Z*	2	4
ρ (calcd) (Mg m^−3^)	2.246	2.193
μ (mm^−1^)	9.790	9.311
*F*(000)	1246.0	1232
Reflections collected/unique	24773/8572	22718/3354
*R*_int_	0.0553	0.0267
2θ_max_ (°)	55.8	50.0
Goodness-of-fit on *F*^2^	1.052	1.126
*R*1 indices [*I* > 2σ(*I*)]	0.0626	0.0217
*wR*2 indices	0.1743	0.0518

Computer programs: *Blue Ice* (McPhillips *et al.*, 2002 ▸), *XDS* (Kabsch, 1993 ▸), *SHELXT2014* (Sheldrick, 2015*a* ▸) and *SHELXL2018* (Sheldrick, 2015*b* ▸).

**Table 2:** Observed and calculated chemical shifts (ppm) for **1*o*** and their com­parison with calculated *m*-BrC_6_F_4_ organo­amido­platinum(II) com­pounds

F	Observed	Calculated for *o*-BrC_6_F_4_	F	Calculated for *m*-BrC_6_F_4_
F3	−140.6	−140.6	F2	−122.5
F6	−151.2	−151.1	F6	−145.1
F5	−160.9	−163.2	F4	−145.2
F4	−171.1	−174.8	F5	−169.2

**Table 3:** Selected bond lengths (Å) and bond angles (°) for **1*ox***/**1*oy*** and com­parison with **1*p***

Bond	**1*ox***	**1*oy***	**1*p***	Angle	**1*ox***	**1*oy***	**1*p***
Pt—Cl	2.35 (3)	2.323 (7)	2.344 (10)	Cl—Pt—N(amide)	177.5 (9)	175.8 (4)	176.17 (9)
Pt—Br	2.534 (16)	2.62 (3)	–	N(amide)—Pt—N(Et_2_)	84.2 (4)	83.5 (4)	82.65 (12)
Pt—N(amide)	1.993 (11)	2.006 (11)	2.006 (3)	N(amide)—Pt—N(py)	91.6 (4)	93.2 (4)	93.27 (12)
Pt—N(Et_2_)	2.087 (10)	2.076 (9)	2.074 (3)	Cl—Pt—N(Et_2_)	93.3 (8)	92.3 (4)	93.98 (9)
Pt—N(py)	2.034 (9)	2.026 (9)	2.013 (3)	Cl—Pt—N(py)	90.9 (8)	90.9 (4)	90.25 (8)
N(amide)—C(C_6_F_4_)	1.383 (19)	1.362 (19)	1.354 (4)	N(Et_2_)—Pt—N(py)	174.8 (4)	173.8 (4)	173.53 (12)
