# Supplementary material for: Syn- and anti-rotamers of the ortho-stereoisomer [Pt{(o-BrC6F4)N(CH2)2NEt2}Cl(py)]
Source: Acta Crystallogr C Struct Chem. 2025 Aug 21;81(Pt 9):513–8. doi: 10.1107/S2053229625006837 (PMC12406265; doi:10.1107/S2053229625006837)
Supplement: Supplementary file 3 [file c-81-00513-sup3.pdf]

Table of contents

Figure S1. The numbering system of the F of the polyfluoroaryl ring in *para*-, *ortho*-, and *meta*-bromo organoamidoplatinum(II) compounds.
Scheme S1. Proposed reaction pathway for the formation of **1*o*** by CO_2 elimination reaction.
Scheme S2. The resonance structures of the polyfluoroaryl ring and delocalisation of the negative charge in the Meisenheimer intermediate, showing the formation of **1*p*** on top and formation of **1*o***(anti) **1*o***(syn) below.
Figure S2. ^{19}F NMR spectrum of [Pt{(*o*-BrC_6F_4)$NCH_2CH_2NEt_2$}Cl(py)] in $(CD_3)_2CO$.
Figure S3. ^{1}H NMR spectrum of [Pt{(*o*-BrC_6F_4)$CH_2CH_2NEt_2$}Cl(py)] in $(CD_3)_2CO$.

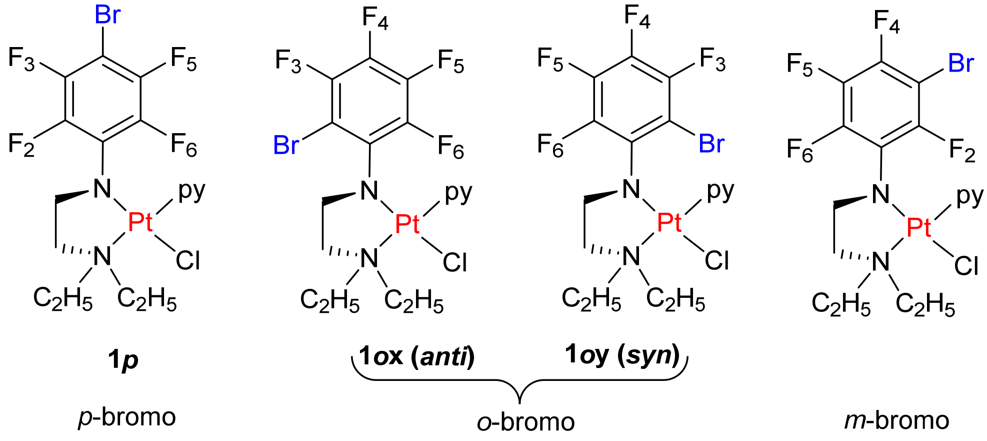

Figure S1 The numbering system of the F of the polyfluoroaryl ring in *para*-, *ortho*-, and *meta*-bromo organoamidoplatinum(II) compounds.

Scheme S1 Proposed reaction pathway for the formation of **1*o*** by CO_2 elimination reaction

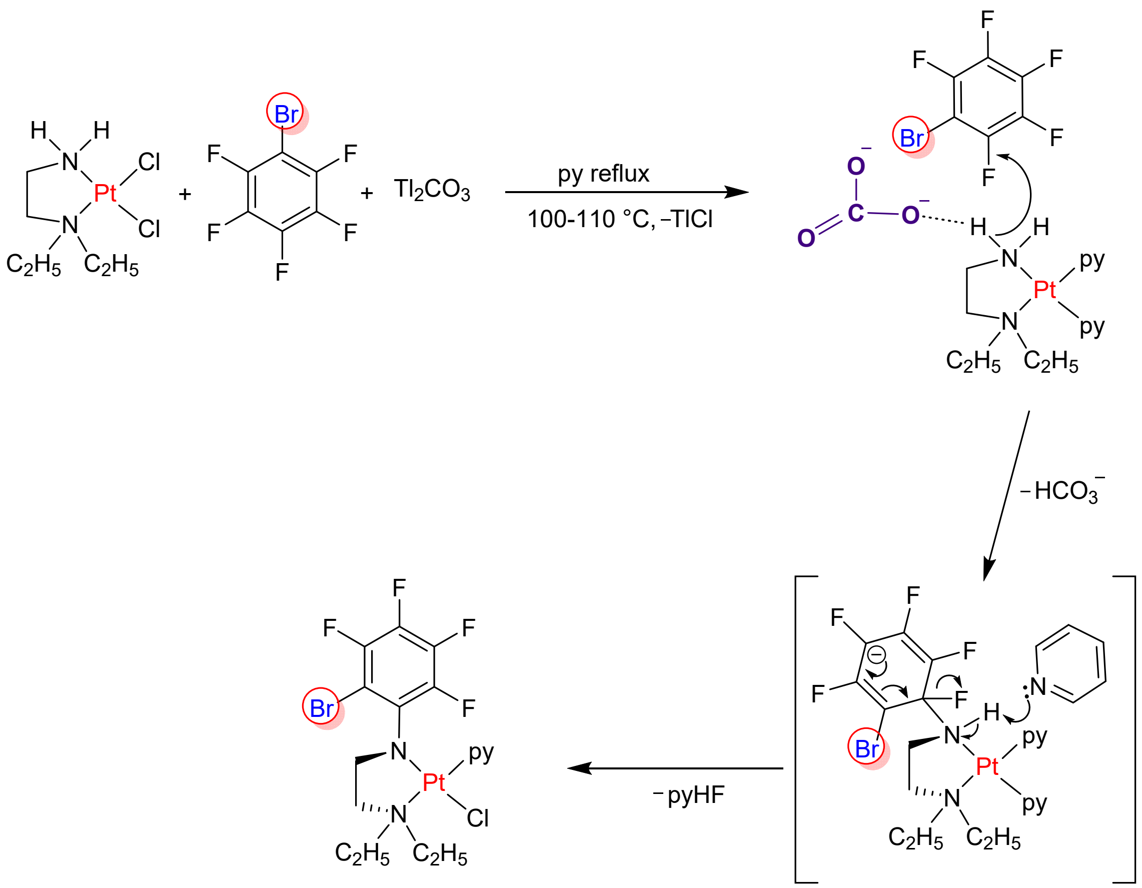

$$2\ pyHF + Tl_2CO_3 \longrightarrow TlF + H_2O + CO_2$$

Scheme S2. The resonating structures of the polyfluoroaryl ring and delocalisation of the negative charge in the Meisenheimer intermediate, showing the formation of **1*p*** on top and formation of **1*o***(anti) **1*o***(syn) below.

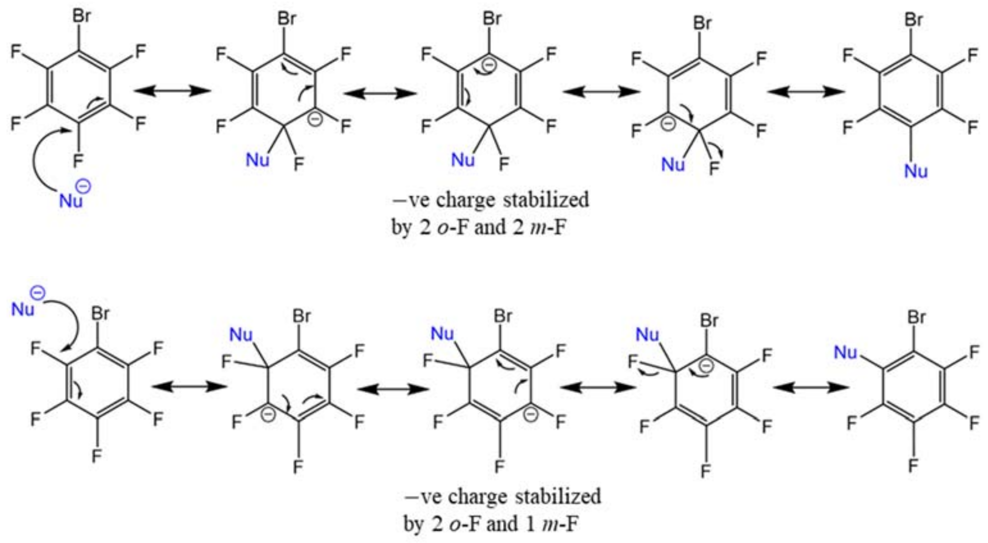

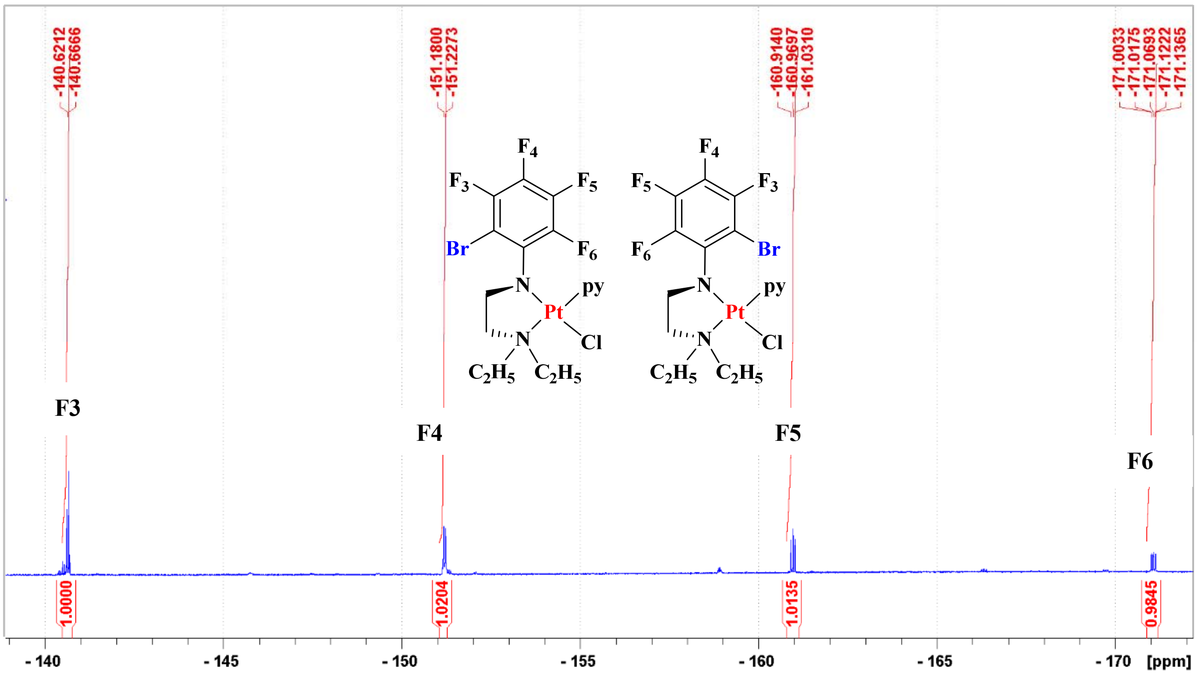

Figure S2. ^{19}F NMR spectrum of $[Pt\{(o\text{-}BrC_6F_4)NCH_2CH_2NEt_2\}Cl(py)]$ in $(CD_3)_2CO$ showing **1ox** and **1oy** in a 1:1 ratio.

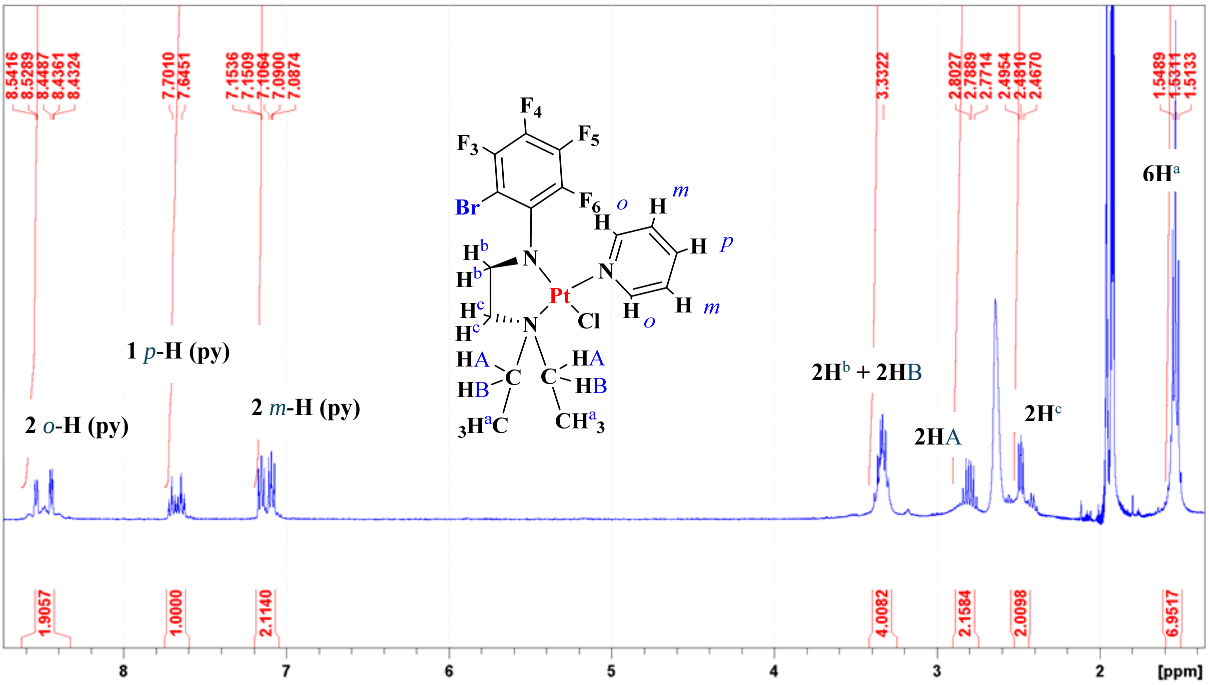

Figure S3. ^{1}H NMR spectrum of $[Pt\{(o\text{-}BrC_6F_4)CH_2CH_2NEt_2\}Cl(py)]$ in $(CD_3)_2CO$ showing **1ox** and **1oy** in a 1:1 ratio.
